# ﻿Further notes on the Afrotropical genus *Festucula* Simon, 1901 (Araneae, Salticidae)

**DOI:** 10.3897/zookeys.1185.110365

**Published:** 2023-12-01

**Authors:** Galina N. Azarkina, Stefan H. Foord

**Affiliations:** 1 Laboratory of Systematics of Invertebrate Animals, Institute of Systematics and Ecology of Animals, Siberian Branch of the Russian Academy of Sciences, Frunze Street 11, Novosibirsk 630091, Russia Institute of Systematics and Ecology of Animals, Siberian Branch of the Russian Academy of Sciences Novosibirsk Russia; 2 SARChI Chair on Biodiversity Value and Change in the Vhembe Biosphere Reserve, Centre for Invasion Biology, Faculty of Science, Engineering and Agriculture, University of Venda, Private Bag X5050, Thohoyandou, Limpopo 0950, South Africa University of Venda Thohoyandou South Africa

**Keywords:** Africa, Botswana, first female, Guinea, jumping spiders, new species

## Abstract

Notes on four *Festucula* species are provided. One species, *F.botswana***sp. nov.**, is described as new to science (♀, Botswana). The name *F.monticola* is revalidated and the male of this species is assigned. The female of *F.lawrencei* is described for the first time. A new record of *F.leroyae* is provided.

## ﻿Introduction

The genus *Festucula* has been revised twice (Wesołowska 1992; Azarkina and Foord 2014). Currently, eight species are included in the genus (WSC 2023). Members of this genus have a strong association with grasses. Some of the species are sympatric, for example, *F.australis* Lawrence, 1927 and *F.lawrencei* Lessert, 1933 (both found in Angola), *F.leroyae* Azarkina & Foord, 2014 and *F.robustus* Azarkina & Foord, 2014 (co-occur in South Africa) (Azarkina and Foord 2014).

Material from a newly studied collection shows that *F.monticola* Berland & Millot, 1941 is a valid species; the female of this species is redescribed. Males, previously associated with *F.lineata* Simon, 1901, belong to *F.monticola* (but see comments under *F.monticola*, Discussion and conclusion). The female of *Festuculalawrencei* is described for the first time, while the male previously known only from the holotype is redescribed. Additionally, a single female of *Festucula* from northern Botswana is diagnosed and described as a new species, *Festuculabotswana* sp. nov. New records of *F.leroyae* in the north of Botswana are provided. All records are mapped.

## ﻿Material and methods

The specimens used in this study are kept in the following collections (curator names are in parentheses):

**NCA** National Collection of Arachnida, Pretoria, South Africa (P. Marais and R. Lyle)

**PCARS** Personal collection of Anthony Russell-Smith, Sittingbourne, UK

**SMF**Senckenberg Natural History Museum, Frankfurt am Main, Germany (P. Jäger)

A total of 13 specimens were examined. Specimens were studied in 70% ethanol and a description of their colouration refers to that of preserved specimens. All drawings were made with the aid of a reticular eyepiece attached to an MBS–10 stereomicroscope at ISEA. Photographs of preserved specimens were taken with a Canon EOS 550D camera attached to a Zeiss Stemi 2000–C stereomicroscope at ISEA. The epigynes were detached and macerated in 10% KOH overnight. After the photos were taken and drawings were made, dissected parts were stored in microvials with the specimens. The drawings were edited in Adobe Photoshop CS5.

The abbreviations used in the figures and text are as follows:

**AG** accessory glands;

**AME** anterior median eyes;

**ap** apical;

**CD** copulatory ducts;

**d** dorsal;

**ED** epigynal depression;

**Fm** femur;

**MS** median septum;

**Mt** metatarsus;

**PLE** posterior median eyes;

**pr** prolateral;

**Pt** patella;

**Tb** tibia;

**v** ventral.

The sequence of leg segments in measurement data is as follows: femur + patella + tibia + metatarsus + tarsus (total). All measurements are in millimetres (mm). Leg setation follows Ono (1988). Terminology follows Azarkina and Foord (2014). The distribution maps were produced using the online mapping software SimpleMappr (Shorthouse 2010).

## ﻿Results

### ﻿Family Salticidae Blackwall, 1841

**Subfamily Salticinae Blackwall, 1841**

**Tribe Chrysillini Simon, 1901**

#### ﻿Genus Festucula

Taxon classificationAnimaliaAraneaeSalticidae

Simon, 1901

8C761455-7F4D-5F42-90E3-C23DD8154AF9

Festucula Simon, 1901: 607.

##### Type species.

*Festuculavermiformis* Simon, 1901: by original designation.

##### Diagnosis, description and distribution.

See Azarkina and Foord (2014).

#### ﻿ Festucula botswana sp. nov.

Taxon classificationAnimaliaAraneaeSalticidae

7061392B-A393-5D6B-BE96-61735CA81ECC

https://zoobank.org/47EFE4C7-7707-42F2-92BB-5A8BED73AEE2

Figs 1–3 , 4–7 , 29 , 30

##### Type material.

***Holotype***: Botswana • ♀; Okavango swamps, c. -19.42, 22.97, on water surface, near plants, 28.VI.1979, A. Morley, B. Taylor leg.; NCA 83/496.

##### Diagnosis.

The epigyne of *Festuculabotswana* sp. nov. is similar to that of *F.festuculaeformis* (Lessert, 1925) and *F.haddadi* Azarkina & Foord, 2014, but differs from them in having longer and thinner accessory glands’ copulatory ducts (shorter in former species, cf. Fig. 2 and Azarkina and Foord 2014: figs 52, 68).

##### Etymology.

This species is named after the country of the type locality.

##### Description.

**Figures 1–3.**
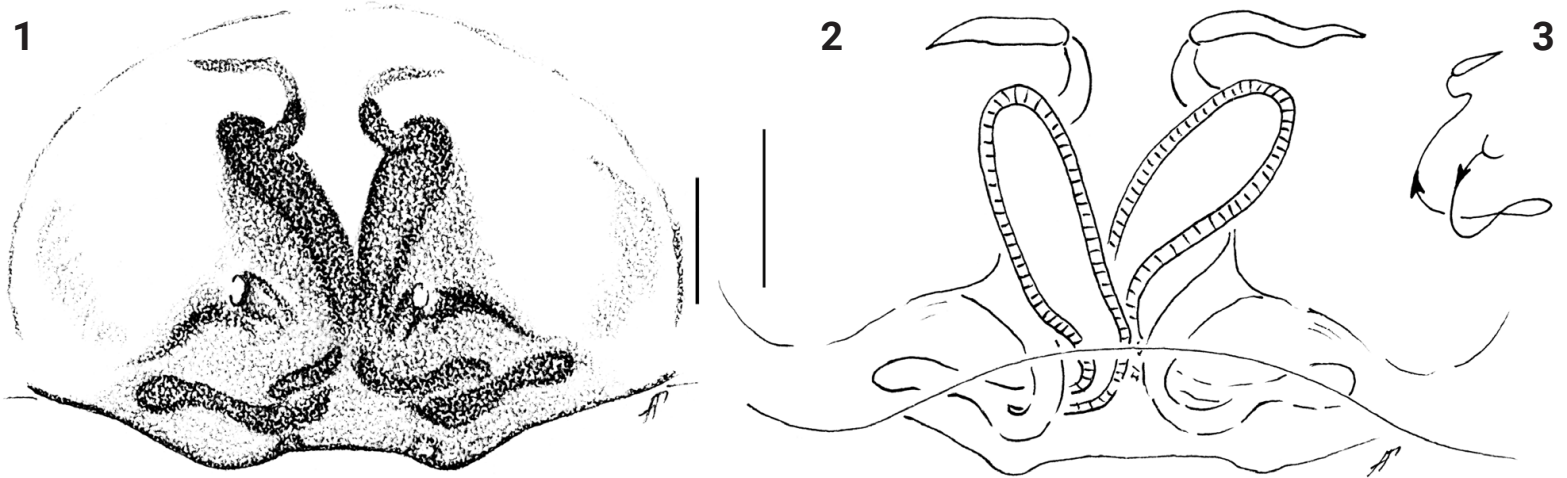
*Festuculabotswana* sp. nov., holotype **1** epigyne, ventral view **2** vulva, dorsal view **3** diagrammatic course of the insemination ducts. Scale bars: 0.1 mm.

**Figures 4–7.**
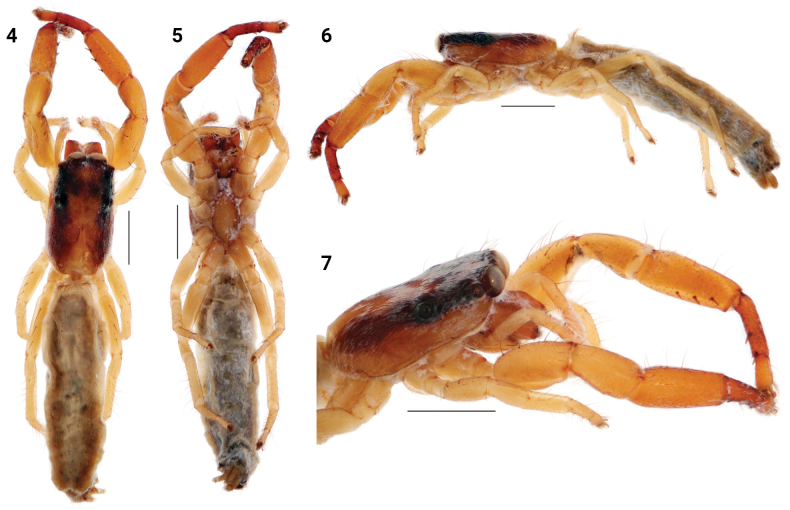
*Festuculabotswana* sp. nov., holotype **4** habitus, dorsal view **5** same, ventral view **6** same, lateral view **7** same, latero-frontal view. Scale bars: 1 mm.

**Female.** Total length 6.40. Carapace 2.05 long, 1.25 width. Abdomen 4.30 long, 1.10 width. Ocular area 0.85 long, 1.00 wide anteriorly, 1.10 wide posteriorly. Cheliceral length 0.65. Clypeal height 0.05. Hight at PLE 0.50. Diameter of AME 0.35. Length of leg segments: I 1.10 + 0.75 + 1.10 + 0.70 + 0.40 (4.05). II 0.85 + 0.50 + 0.55 + 0.50 + 0.35 (2.75). III. 0.75 + 0.30 + 0.50 + 0.55 + 0.40 (2.50). IV 1.25 + 0.50 + 1.00 + 0.80 + 0.50 (4.05). Leg setation: I: Tbpr 1-1-1, Mt v 2-2 ap. II: Mt v 2-2 ap. Colouration (in alcohol, Figs 4–7). Carapace brown, with a broad light brown longitudinal band medially and pair of broad light brown bands marginally. Ocular area brown, with two dark-brown patches in the middle, with black patches around eyes. Sternum yellow. Labium and endites yellow, pale apically. Chelicerae brown-yellow. Clypeus and cheeks brown-yellow, covered with white setae. Carapace stridulatory organs with 7 seta-bearing tubercles. Abdomen light brown, with one broad whitish-yellow longitudinal band medially and two whitish-yellow longitudinal bands laterally. Venter brownish-grey, covered with white short setae. Spinnerets brownish-yellow. Book-lung covers yellow. All legs yellow. Legs I long and robust, yellow-brown. Femora I dark-brown prolatero-apically; patellae I dark-brown prolaterally. Metatarsi and tarsi I brown. Palps yellow. Epigyne and vulva as in Figs 1–3: wider than long in about 1.5 times. Copulatory openings located almost in a middle part, small and roundish. Fertilization ducts visible through integument. Copulatory ducts long, connected to long and club-shaped accessory glands pointed laterally. Spermathecae long and sub-vertical, with fertilization ducts located on apical part.

##### Distribution.

Known only from type locality in Botswana (Fig. 29).

#### ﻿ Festucula lawrencei

Taxon classificationAnimaliaAraneaeSalticidae

Lessert, 1933

3DA25A3A-6C0C-5547-B955-8062447BC49C

Figs 8–13 , 14–21 , 29 , 30

Festucula lawrencei Lessert, 1933: 152, fig. 72 (Type ♂, Angola: Santo Amaro, MRAC—examined).
F. lawrencei : Wesołowska 1992: 50, figs 26–27; Azarkina and Foord 2014: 365, figs 77–87.

##### Material examined.

Botswana • 1♂2♀; North-West District, nr Maun, Manxunyane Lagoon, -19.90, 23.37, floodplain grassland, sweeping; 21.IX.1976; A. Russell-Smith leg.; PCARS.

##### Diagnosis.

The epigyne and vulva of *F.lawrencei* resemble that of *F.haddadi* and *F.leroyae*, but differ from *F.haddadi* in having longer and thinner copulatory ducts (cf. Figs 41 and 43); from *F.leroyae* in having smaller copulatory openings (cf. Figs 42 and 44). For male’s diagnosis see Azarkina and Foord 2014: 365.

##### Description.

**Figures 8–13.**
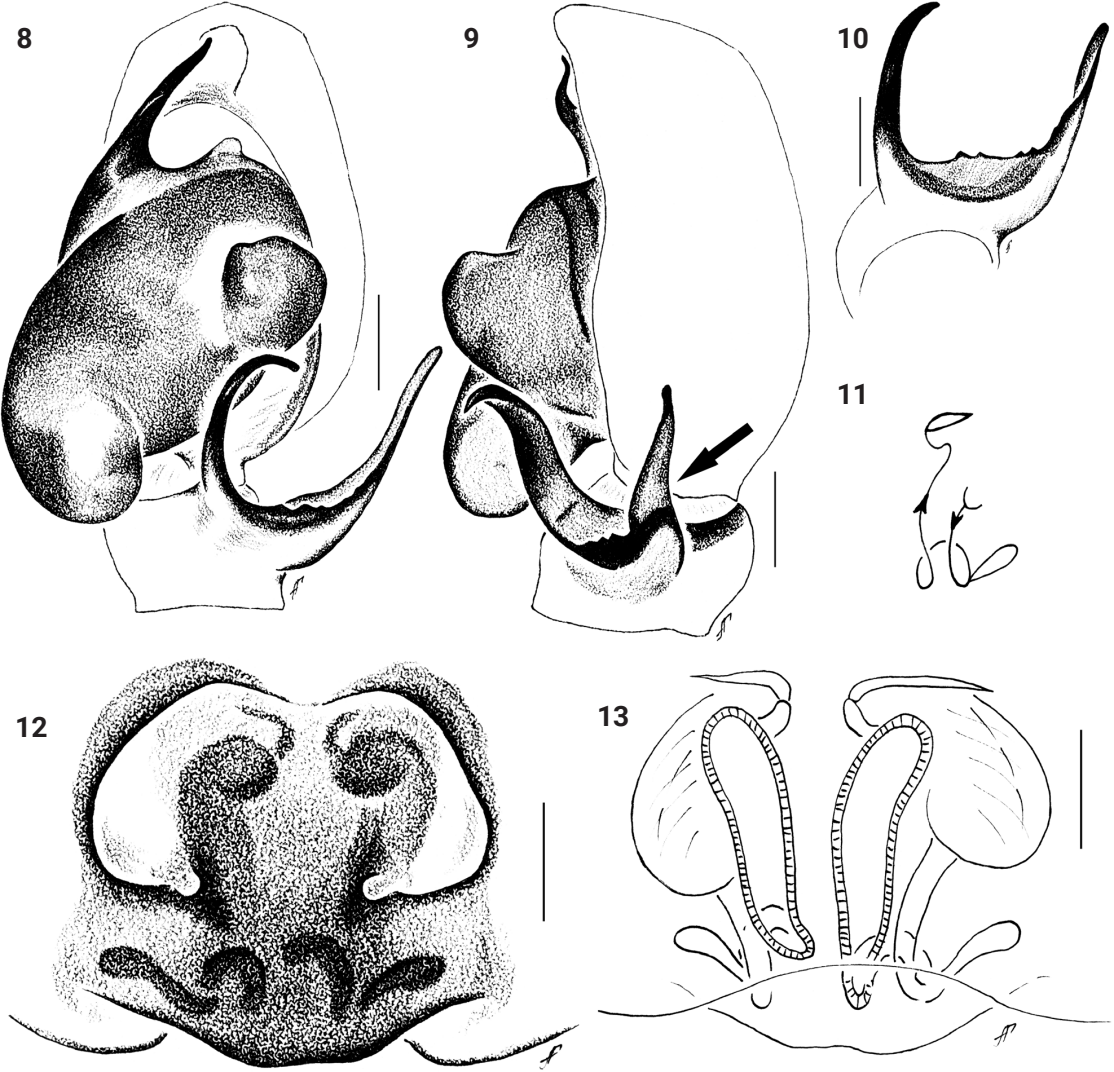
*Festuculalawrencei***8** male palp, ventral view **9** same, retrolateral view **10** tibial apophysis, ventro-retrolateral view **11** diagrammatic course of the insemination ducts **12** epigyne, ventral view **13** vulva, dorsal view. Scale bars: 0.1 mm.

**Figures 14–21.**
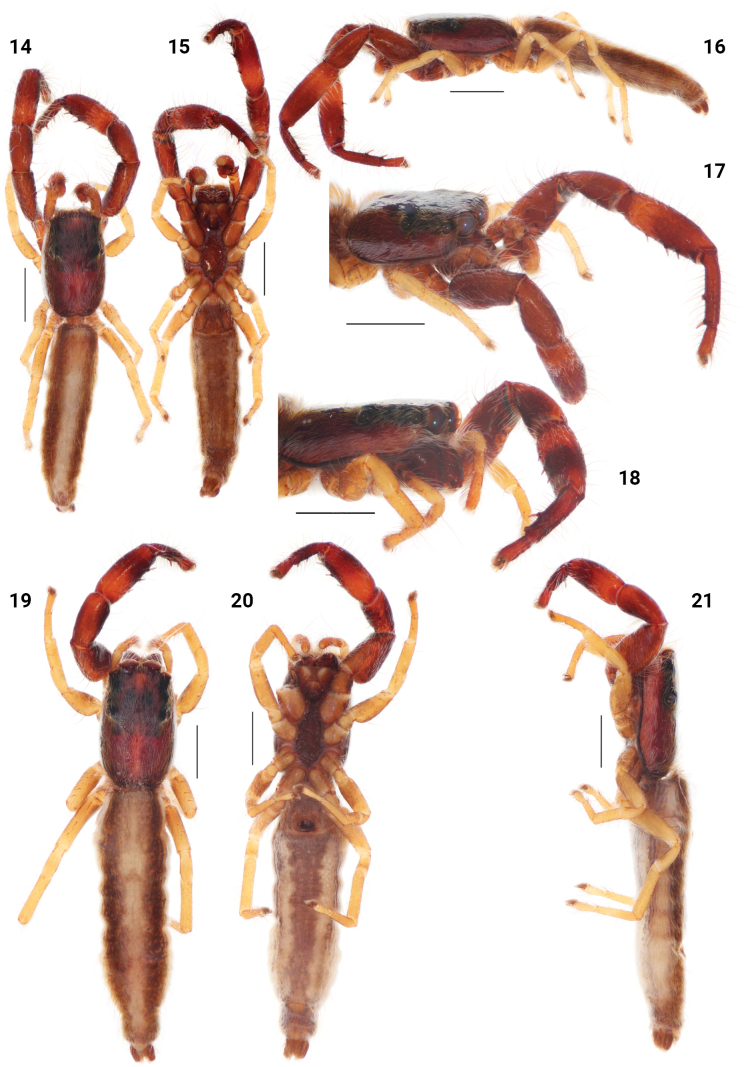
*Festuculalawrencei***14** male habitus, dorsal view **15** same, ventral view **16** same, lateral view **17** same, latero-frontal view **18** female habitus, latero-frontal view **19** same, dorsal view **20** same, ventral view **21** same, lateral view. Scale bars: 1 mm.

**Male.** Total length 5.80. Carapace 2.00 long, 1.25 wide. Abdomen 3.70 long, 0.90 wide. Ocular area 0.85 long, 0.95 wide anteriorly, 1.05 wide posteriorly. Cheliceral length 0.50. Clypeal height 0.05. Height at PLE 0.60. Diameter of AME 0.30. Length of leg segments: I 1.50 + 1.00 + 1.50 + 1.00 + 0.45 (4.45). II 0.80 + 0.35 + 0.50 + 0.45 + 0.30 (2.40). III 0.75 + 0.35 + 0.50 + 0.50 + 0.30 (2.40). IV 1.10 + 0.50 + 0.75 + 0.70 + 0.40 (3.45). Leg setation: I: Tbpr 1-1-1, Mt v 2-2 ap. Colouration (in alcohol, Figs 14–17). Carapace brown, covered with brown setae. with broad longitudinal bands of white setae medially and laterally. Clypeus brown, covered with white setae. Sternum brown. Endites and labium brown, pale apically. Chelicerae brown. Carapace stridulatory organs with 8 seta-bearing tubercles. Abdomen brown dorsally, with broad yellow-white longitudinal band medially, with narrow yellowish longitudinal bands laterally. Venter brown. Spinnerets brown. Book-lung covers yellow-brown. Legs I robust, long, brown. Remaining legs yellow. Palps brown. Palpal structure as in Figs 8–10: tegulum with prolateral basal and retrolateral median lobes. Embolus pointed apically at 45° from tegulum, with small bump on tegulum near embolic base. Tibia short. Tibial apophyses thin, curved, with multiple small teeth between lateral and ventral aphophyses. Lateral tibial apophysis concave.

**Female.** Total length 7.30–9.10. Carapace 2.35–2.40 long, 1.45–1.50 wide. Abdomen 5.00–6.70 long, 1.45–1.50 wide. Ocular area 1.00–1.10 long, 1.15–1.20 wide anteriorly, 1.25–1.30 wide posteriorly. Cheliceral length 0.60–0.80. Clypeal height 0.05. Height at PLE 0.70. Diameter of AME 0.40. Length of leg segments (smallest female): I 1.50 + 1.00 + 1.30 + 0.90 + 0.45 (5.15). II 1.00 + 0.50 + 0.60 + 0.55 + 0.35 (3.00). III 0.90 + 0.45 + 0.50 + 0.70 + 0.40 (2.95). IV 1.30 + 0.70 + 1.00 + 0.90 + 0.50 (4.40). Leg setation: I: Tbpr 1-1-1, Mt v 2-2 ap. II: Mt v 2-2 ap. Colouration (in alcohol, Figs 18–21).Similar to that of male, but legs II–IV and palps brownish-yellow. Epigyne and vulva as in Figs 11–13: Epigyne with two small round copulatory openings located in posterior half submedially. Copulatory ducts and accessory glands long and thin, spermathecae long and thin. Fertilization ducts located apically.

##### Distribution.

Angola and Botswana (Fig. 29).

##### Comments.

Palps of specimens from Botswana differ slightly from palps of the type specimen: the lateral tibial apophysis is less concave than the type (cf. Fig. 9, arrowed and Azarkina and Foord 2014: fig. 78). The inner edge of the LTA has multiple small teeth in specimen from Botswana vs. one big tooth in type specimen (cf. Fig. 10 and Azarkina and Foord 2014: figs 80, 81).

#### ﻿ Festucula monticola

Taxon classificationAnimaliaAraneaeSalticidae

Berland & Millot, 1941

2FA31067-9C27-50C8-A8A6-E8F23049F5C6

Figs 22–24 , 25–28 , 29 , 30

Festucula monticola Berland & Millot, 1941: 345, fig. 48 (Type ♀, Guinea: Dalaba, MNHN—not examined, probably lost).
F. monticola : Wesołowska 1992: 45 (Nomen dubium); Azarkina and Foord 2014: 374 (Nomen dubium).
F. festuculaeformis : Wesołowska and Edwards 2012: 746, figs 44–45 (misidentified).
F. lineata : Fage 1923: 299, fig. 1; Azarkina and Foord 2014: 369 (misidentified).

##### Material examined.

Guinea • 1♀; Nzérékoré Region, Prefecture Lola, Mount Nimba, c. 7.67, -8.40; M. Lamotte leg.; SMF 72421 • 1♀; same but 11.XII.1956, SMF 72420.

##### Diagnosis.

The female of this species resembles *F.australis* and *F.vermiformis* in epigyne and vulva form, but differs from *F.australis* by having larger epigynal depressions (cf. Figs 30 and 32), and from *F.vermiformis* in the accessory gland located closer to epigastric furrow (cf. Figs 33 and 35). Male resembles that of *F.australis* (for male’s diagnosis see Azarkina and Foord 2014: 369, sub *F.lineata* sensu Fage 1923).

##### Description.

**Figures 22–24.**
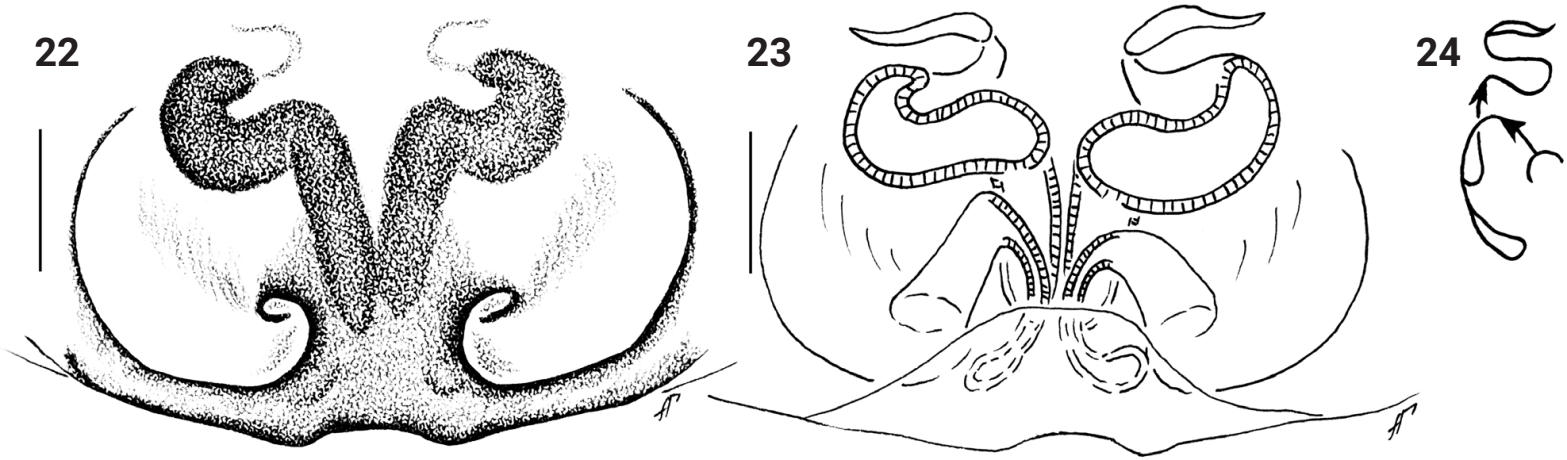
*Festuculamonticola***22** epigyne, ventral view **23** vulva, dorsal view **24** diagrammatic course of the insemination ducts. Scale bars: 0.1 mm.

**Figures 25–28.**
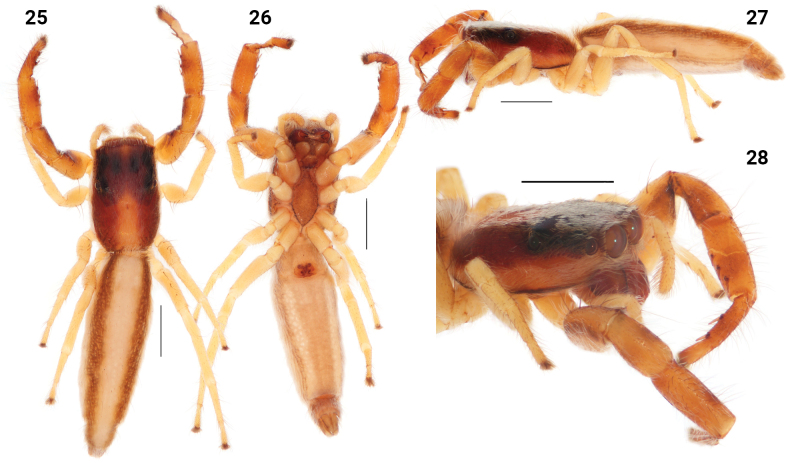
*Festuculamonticola***25** habitus, dorsal view **26** same, ventral view **27** same, lateral view **28** same, latero-frontal view. Scale bars: 1 mm.

**Female.** Total length 5.80. Carapace 2.10 long, 1.40 wide. Abdomen 3.70 long, 1.30 wide. Ocular area 0.85 long, 1.15 wide anteriorly, 1.20 wide posteriorly. Cheliceral length 0.60. Clypeal height 0.05. Hight at PLE 0.70. Diameter of AME 0.30. Length of leg segments: I 1.10 + 0.80 + 0.95 + 0.65 + 0.40 (3.90). II 0.80 + 0.45 + 0.55 + 0.50 + 0.40 (2.70). III 0.80 + 0.45 + 0.55 + 0.50 + 0.40 (2.70). IV 1.30 + 0.60 + 0.90 + 0.90 + 0.45 (4.15). Leg setation: I: Fm d 0-1-1-2, Tbpr 1-1-1, Mt v 2-2 ap. II: Fm d 0-1-1-1, Mt v 2-2 ap. III: Fm d 0-1-1-1. IV: Fm d 0-1-1-1. Colouration (in alcohol, Figs 25–28). Carapace brown, with a broad yellow longitudinal band medially and pair of broad yellow bands marginally. The ocular area is brown, with two dark-brown patches in the middle part, with black patches around eyes. Sternum yellow-brown. Labium and endites yellow-brown, pale apically. Chelicerae are dark-brown. Clypeus and cheeks yellow, covered with white setae. Carapace stridulatory organs with 8 seta-bearing tubercles. Abdomen brown, with one broad yellow longitudinal band medially and two yellow longitudinal bands laterally. Venter white-yellow, with dark yellow longitudinal band, tinged with brown medially; with two narrow longitudinal brownish bands laterally. Spinnerets brownish-yellow. Book-lung covers brownish. Legs I long and robust, brownish. Femora I dark-brown prolatero-apically; patellae I dark-brown prolaterally. Tibiae and metatarsi I brown in apical half prolaterally. Remaining legs yellow. Palps yellow. Epigyne and vulva as in Figs 22–24: 1.3 times wider than long. Copulatory openings located in posterior half. Fertilization ducts visible through integument. Copulatory ducts broad, pointed anteriorly. Accessory glands club-shaped, pointed posteriorly. Spermathecae Г-shaped, fertilization ducts located on apical part.

##### Distribution.

**Figure 29.**
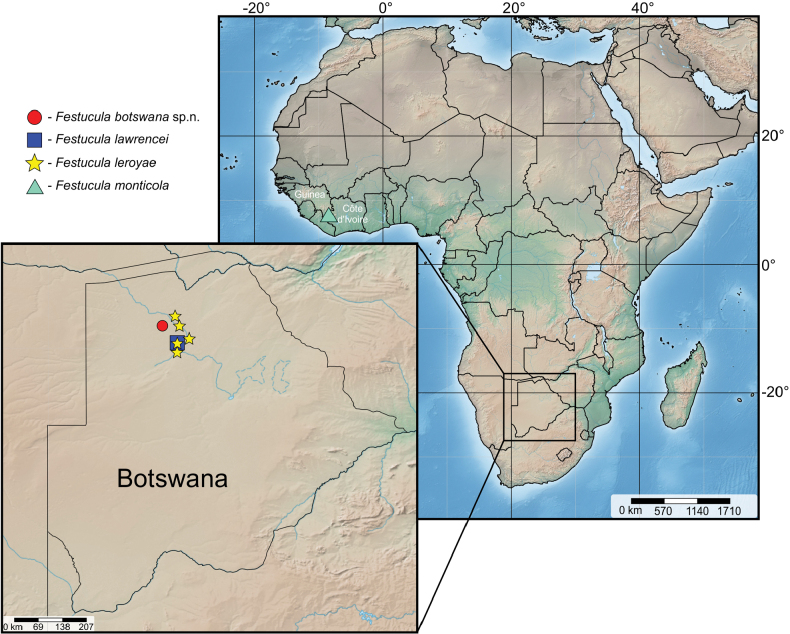
New data of distribution of four *Festucula* species in Africa.

Guinea, Côte d’Ivoire (Fig. 29).

##### Comments.

Due to the possible loss of the type specimens of *F.lineata* and *F.monticola* (Wesołowska 1992; Azarkina and Foord 2014), the specific status of these species remains uncertain. Wesołowska (1992) considered *F.lineata* and *F.monticola* as *nomina dubia*. *Festuculalineata* was described from Dakar, Senegal and the type specimens are immature (Wesołowska 1992), but Lessert (1933, f. 73) drew the type specimen and at least one female was mature (this female probably lost). The type of *F.monticola* was described from Dalaba, Guinea (Berland and Millot 1941). Later Fage (1923) described a male of *F.lineata* from Kerouane, Guinea. Based on a single male from Bouaké, Côte d’Ivoire and figures of a male from Guinea (Fage 1923) Azarkina and Foord (2014) revalidated *F.lineata* (sensu Fage, 1923). The description of the male as *F.lineata*, by Fage, seems erroneous. Based on new material from Mt. Nimba in Guinea we conclude that the localities of the Fage (1923) and Azarkina and Foord (2014) males falls within *F.monticola* distribution range of and are therefore *F.monticola* instead of *F.lineata*. We therefore revalidated the status of *F.monticola*, redescribed the female and assigned males from Guinea (Fage 1923) and Côte d’Ivoire (Azarkina and Foord 2014) as males of *F.monticola* instead of *F.lineata*.

###### ﻿New record

#### ﻿ Festucula leroyae

Taxon classificationAnimaliaAraneaeSalticidae

Azarkina & Foord, 2014

62EE7731-821A-5CDC-A1EA-BE8D8F357D85

Fig. 30

##### Material examined.

Botswana • North-West District • 1♂1♀; nr Maun, Manxunyane Lagoon, -19.90, 23.37, floodplain grassland, sweeping; 21.IX.1976; A. Russell-Smith leg.; PCARS • 1♀ nr Maun, Moshi Bridge, -20.10, 23.35, floodplain grassland; 10.III.1976; A. Russell-Smith leg.; PCARS • 1♂; dried out Lagoon nr Maphaneng Pan, -19.41, 23.42, sweeping; 27.II.1976; A. Russell-Smith leg.; PCARS • 1♀; Okavango, Shorobe Lagoon, -19.75, 23.67, floodplain grassland, sweeping; 28.VIII.1975; A. Russell-Smith leg.; PCARS • 1♂2♀; Mboma Island, -19.17, 23.30, *Miscanthidium* grassland field layer; 7.X.1975; A. Russell-Smith leg. (PCARS).

**Figures 30–47.**
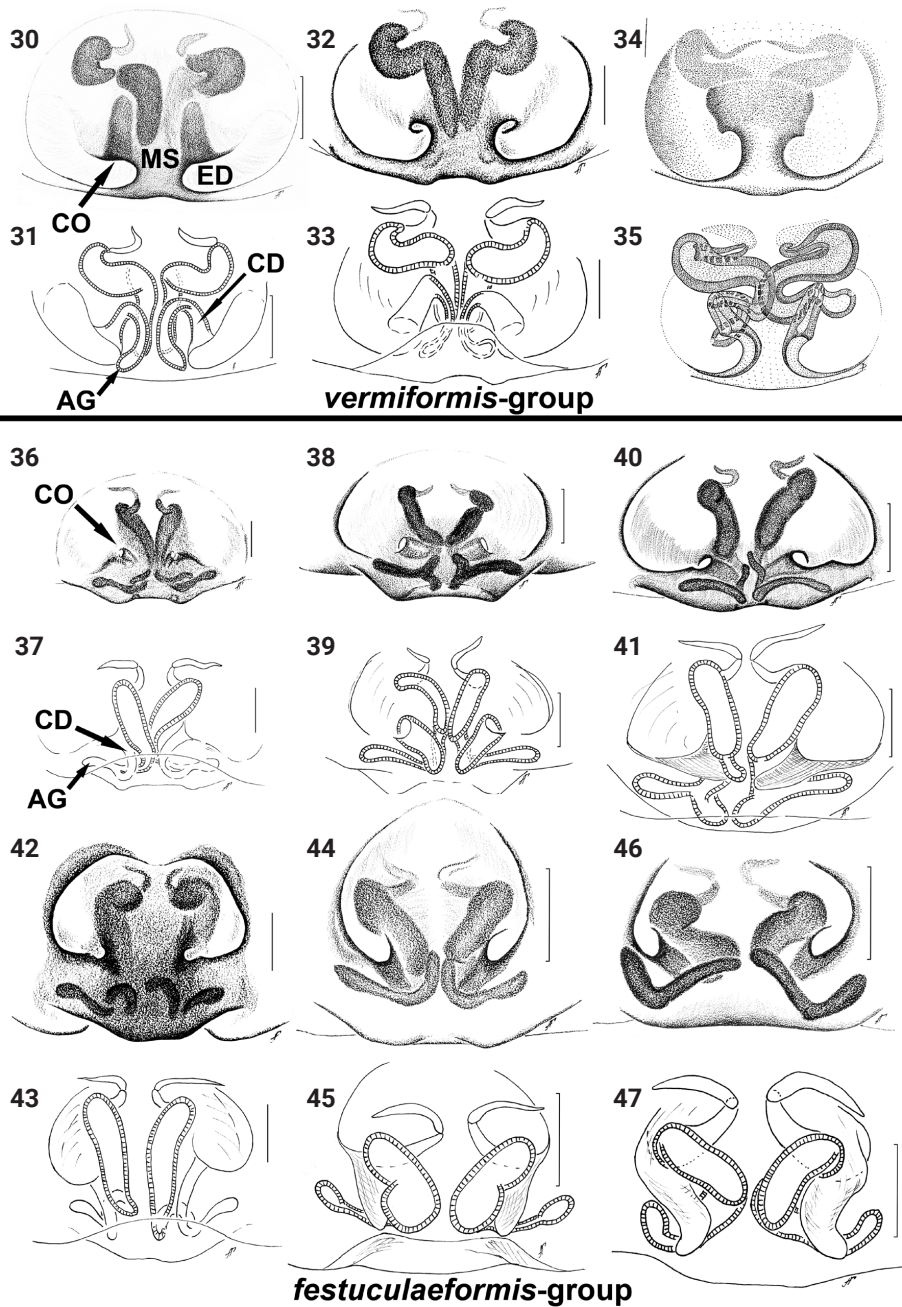
*Festucula* spp., females **30, 31***F.australis* (from Azarkina and Foord 2014) **32, 33***F.monticola***34, 35***F.vermiformis* (from Prószyński 1987) **36, 37***F.botswana* sp. nov. **38, 39***F.festuculaeformis* (from Azarkina and Foord 2014) **40, 41***F.haddadi* (from Azarkina and Foord 2014) **42, 43***F.lawrencei***44, 45***F.leroyae* (from Azarkina and Foord 2014) **46, 47***F.robustus* (from Azarkina and Foord 2014): **30, 32, 34, 36, 38, 40, 42, 44, 46** epigyne, ventral view **31, 33, 35, 37, 39, 41, 43, 45, 47** vulva, dorsal view. Scale bars: 0.1 mm.

## ﻿Discussion and conclusion

**Figures 48–55.**
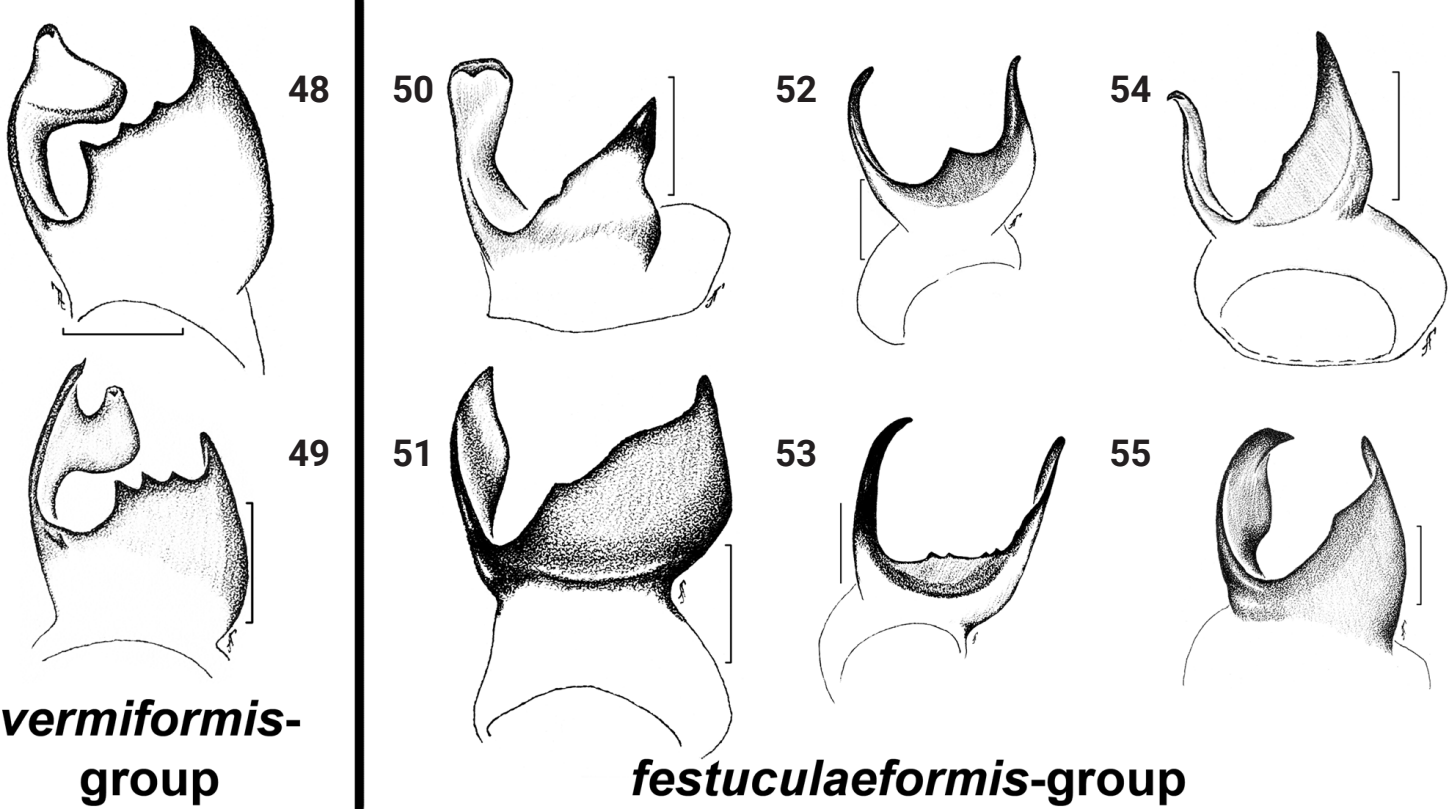
*Festucula* spp., males, tibial apophyses **48***F.australis*, retrolateral-apical view (from Azarkina and Foord 2014) **49***F.monticola*, ventro-retrolateral view (from Azarkina and Foord 2014, sub *F.lineata*) **50***F.festuculaeformis*, retrolateral-apical view (from Azarkina and Foord 2014) **51***F.haddadi*, ventro-retrolateral view (from Azarkina and Foord 2014) **52, 53***F.lawrencei*, **52** retrolateral-apical view (from Azarkina and Foord 2014) **53** ventro-retrolateral view **54***F.leroyae*, retrolateral-apical view (from Azarkina and Foord 2014) **55***F.robustus*, retrolateral-apical view (from Azarkina and Foord 2014). Scale bars: 0.1 mm.

*Festucula* consists of eight valid species; one species, *F.monticola* was considered *nomen dubium* (WSC 2023). Females of some species are difficult to distinguish because of similarity in epigyne structure. Based on the structure of the copulatory organs, all species of *Festucula* can be divided into two species-groups:

- ***vermiformis* -group**: (1) epigyne with median septum (MS), copulatory openings (CO) on lateral sides of median septum in oval and large epigynal depressions (ED), located close to middle part of epigyne; lower rim of epigynal depressions is close to epigastric furrow (Figs 30, 32, 34); (2) copulatory ducts (CD) directed upward, then bent and directed downward; (3) accessory glands (AG) directed downward (Figs 31, 33, 35); (4) tip of the ventral lateral tibial apophysis (LTA) swollen or bifurcated; and (5) inner edge of the LTA with well-marked teeth (Figs 48–49). This group includes three species: *F.australis*, *F.monticola* and *F.vermiformis*;
- ***festuculaeformis* -group**: (1) epigyne without median septum and copulatory openings not lying in epigynal depression, located close to middle or lateral part of epigyne; lower rim of CO close to the median part of epigyne (Figs 36, 38, 40, 42, 44, 46); (2) CD directed downward; (3) AG directed laterally or upward (Figs 37, 39, 41, 43, 45, 47); (4) tip of ventral LTA pointed; and (5) inner edge of the LTA with one tooth or numerous small teeth (Figs 50–55). This group includes six species: *F.botswana*, *F.festuculaeformis*, *F.haddadi*, *F.lawrencei*, *F.leroyae* and *F.robustus*.

We excluded *F.lineata* from this classification as Lessert’s drawing is too schematic, and we are unable to see the structure of the vulva. Females of *F.leroyae* and *F.robustus* differ from other members of *festuculaeformis*-group in their larger COs, which are located laterally on the epigynal plate, CDs going downward at almost 90° vs 45° in other members of this group except *F.lawrencei*, and AGs going upward at 45° (Figs 44–47) vs almost horizontal in others, except *F.lawrencei*. Females of *F.lawrencei* are intermediate between *F.leroyae*, *F.robustus* and other members of the *festuculaeformis*-group in the structure of the epigyne, with a small CO located in the medially on the epigyne (Figs 42, 43), but the vulva resembles that of *F.leroyae* and *F.robustus*. So, we decided not to establish a new species-group for these two species and include them all in the *festuculaeformis*-group.

*Festuculalawrencei* was the only species known from a single male type specimen. We found both sexes in northern Botswana – the male resembles the type specimen of *F.lawrencei* but differs slightly in the shape of dorsal tibial apophysis and number of teeth on the inner edge of the LTA (Figs 52, 53). Azarkina and Foord (2014) suggested that the number of teeth are species-specific (contra Wesołowska 1992), but it’s probably not. More specimens are required to study intraspecific variation in *F.lawrencei* and other *Festucula* species.

The small number of specimens and schematic drawings of *Festucula* from Western Africa also led to a few taxonomic issues. A single female from Calabar, Nigeria identified as *F.festuculaeformis* by Wesołowska and Edwards (2012), later placed in *F.lineata* by Azarkina and Foord (2014) resembles *F.monticola* and probably belongs to this or a new species. Yet, *F.monticola* might be a junior synonym of *F.vermiformis* (Prószyński 2003). Additional material for *Festucula* from western and northern Africa, preferably both sexes, is required to clarify this issue. Although *F.lineata* is known only based on a type specimen which seems to be lost, we prefer to keep this name valid, because, based on Lessert’s drawing (Lessert 1933: fig. 73), it differs from both *F.monticola* and *F.vermiformis* and might be a separate species. This would require further attention.

The single holotype specimen of *F.botswana* sp. nov. resembles two species, *F.festuculaeformis* from Kenya and Tanzania and *F.haddadi* Azarkina & Foord, 2014 from South Africa, but differs in the vulva structure. We decided to describe this specimen as a new species, but it might be synonymized with one of the abovementioned species. Males and more specimens are required to resolve this uncertainty.

## Supplementary Material

XML Treatment for Festucula

XML Treatment for Festucula botswana

XML Treatment for Festucula lawrencei

XML Treatment for Festucula monticola

XML Treatment for Festucula leroyae

## ﻿Additional information

### Author ORCIDs

Galina N. Azarkina https://orcid.org/0000-0002-9328-3913

Stefan H. Foord https://orcid.org/0000-0002-9195-2562

### Data availability

All of the data that support the findings of this study are available in the main text.
